# Effect of bamboo grass (*Tiliacora triandra*, Diels) pellet supplementation on rumen fermentation characteristics and methane production in Thai native beef cattle

**DOI:** 10.5713/ajas.18.0703

**Published:** 2019-01-04

**Authors:** Chinda Wann, Metha Wanapat, Chaowarit Mapato, Thiwakorn Ampapon, Bi-zhi Huang

**Affiliations:** 1 Tropical Feed Resources Research and Development Center (TROFREC), Department of Animal Science, Faculty of Agriculture, Khon Kaen University, Khon Kaen 40002, Thailand; 2 Yunnan Academy of Grassland and Animal Science, Kunming 650212, Yunnan, China

**Keywords:** Rumen Fermentation, Bamboo-Cass, Rice Straw, Methane Production

## Abstract

**Objective:**

The objective of this study was to investigate the effect of bamboo grass (*Tiliacora triandra*, Diels) pellet (Bamboo-Cass) supplementation on feed intake, nutrient digestibility, rumen microbial population and methane production in Thai native beef cattle.

**Methods:**

Four Thai native beef cattle bulls (190±2 kg) were randomly allotted to four respective dietary treatments in a 4×4 Latin square design. Treatments were the varying levels of Bamboo-Cass supplementation at 0, 50, 100, and 150 g/head/d, respectively. Rice straw was fed *ad libitum* and the concentrate offered at 0.5% of body weight.

**Results:**

Under this experiment, the findings revealed that acetate and butyrate production were decreased (p<0.05), propionate increased (p<0.05), whilst ruminal NH_3_-N concentration was decreased (p<0.05) by supplementation of Bamboo-Cass at 150 g/head/d. Moreover, rice straw intake, and microbial population were linearly increased (p<0.05), while methane production was decreased (p<0.05).

**Conclusion:**

The results from the present study indicate that supplementation of Bamboo-Cass at 150 g/head/d significantly enhanced feed intake, decreased protozoa and increased bacterial population, rumen fermentation efficiency while decreased methane production. Therefore, Bamboo-Cass as a supplement is promising as a rumen enhancer in beef cattle fed on rice straw.

## INTRODUCTION

Greenhouse gases including CH_4_, CO_2_, and N_2_O are emitted from rumen fermentation and results in a loss of dietary energy intake accounting up to 12%. There are many possible CH_4_ mitigation strategies but one of the most promising implementations could be the dietary manipulating option which has been reported in the previous studies [1,2]. There have been numerous reports on the potential use of plant secondary compounds, particularly the use of condensed tannins and crude saponins on rumen microorganisms and the consequent fermentation end-products in mitigating rumen CH_4_ production [3,4].

Bamboo grass (*Tiliacora triandra*, Diels) is a tropical climbing plant with abundant green leaves and yellow flower, belonging to *Menispermaceae* family. It has been known as a local resource plant in sub-tropical and tropical regions, used for medicine, cosmetics, and household products [5,6]. Bamboo grass is rich in phenolic compounds which are useful as a source of bioactive compounds [7]. Previous studies were conducted using this plant to investigate their antioxidant activities, and bioactive role as a medical plant [6,8,9]. Bamboo grass contains plant secondary compounds of condensed tannins (2.3% of dry matter [DM]) and crude saponins (1.3% of DM), and is potential feed resource for use as a strategic supplement in ruminant production [10,11]. However, information on the potential use of this plant in ruminant feeding is not yet available. Therefore, the objective of this study was to determine the effect of bamboo grass pellet (Bamboo-Cass) on feed intake, nutrient digestibility, rumen microbial population, and methane production in Thai beef cattle fed on rice straw.

## MATERIALS AND METHODS

### The experimental area and animals care

This experiment was done at the Ruminant Nutrition and Metabolism Research Center, Tropical Feed Resource Research and Development Center (TROFREC), Department of Animal Science, Faculty of Agriculture, Khon Kaen University (KKU), Khon Kaen, Thailand. All applicable international, national, and/or institutional guidelines for the care and use of animals were followed and allowed by the Khon Kaen University Animal Ethics Committee, ancillary to the Ethic of animal Experimentation of National Research Council of Thailand.

### Animals, feeds and experimental design

All experimental beef cattle were treated with vitamin AD_3_E injection (20 mL/head) and dewormed before imposing the respective treatments. They were raised in individual pens of 3×4 m size where the water and mineral block were always available. Four Thai native beef cattle bulls (190±2 kg) were randomly assigned to receive four dietary treatments in a 4×4 Latin square design and were raised in animal pens with permanent roof. Animals were fed *ad libitum* with rice straw and water supply. The 14% crude protein (CP) of concentrate was formulated and provided to all animals at 0.5% body weight (BW). All animals received four varying levels of Bamboo-Cass supplementation at 0, 50, 100, and 150 g/head/d, respectively. Each of the periods lasted for 21 days, the first 14 days was for adaptation and measurement of intake, while the last 7 days was for sample collections when the experimental animals were moved to the metabolism crates for total collections.

Bamboo grass (*Tiliacora triandra*, Diels) was harvested from fresh leaves from the climbing tree and sun-dried for use in the experiment. Bamboo grass pellets were made by combining bamboo grass (90%), cassava chip (9%), and molasses (1%) (Bamboo-Cass) then sun-dried to achieve at least 85% DM before storage for later feeding to the experimental animals [12].

### Data collection and sampling procedures

All procedures and collection details of the feeds, urine, rumen fluid, blood samples, and rumen methane calculation were done as described by [13]. Animals were fed twice daily at 08:00 hours in the morning and 16:00 hours in the afternoon. Feed offered and refusal feeds were recorded throughout the experimental period for calculating feed intake. Feed and fecal samples were collected during the 7 days feeding period when the animals were in the metabolism crates for analyzing DM, for each period then stored at −20°C for later chemical analysis. The samples were divided into two parts, the first part was analyzed for DM, and the second part kept for analysis of ash, CP, and acid detergent fiber (ADF) [14], neutral detergent fiber (NDF) [15]. The content of condensed tannins of bamboo grass and Bamboo-Cass were analyzed by using the modified vanillin-HCL method [16]. Crude saponins were analyzed by using methanol extraction [17].

Rumen fluid was taken from the middle part of rumen at 0 and 4 hour post morning feeding on the last day of each period prior to morning feeding using a tube connected with the vacuum pump. Rumen fluid was measured immediately for pH (HANNA Instruments HI 8424 microcomputer, Singapore) and strained through four layers cheesecloth. Fluid sample was divided into three parts for continuing analysis. The first 45 mL of rumen fluid sample was collected and kept in plastic bottle to which 5 mL of 1 M H_2_SO_4_ were added for stopping the process of microbial fermentation and then centrifuged with 3,000×g for 10 minutes for volatile fatty acids (VFAs) analysis in the laboratory using high-performance liquid chromatography instruments by water and Novapak model 600E; water mode 1484UV detector; column Novapak C18; column size 3.9×300 mm; mobile phase 10 mM H2PO4 (pH 2.5) [18] and NH_3_-N analysis [14]. The second part (1 mL of rumen fluid) was quickly fixed with 10% of formalin solution (1:9 v/v, rumen fluid:10% formalin) in plastic bottles for measurement of the microbial population. The total direction counts, method of microbial populations were calculated based on the use of a haemacytometer (Boeco, Singapore) [19]. The third part (10 mL of rumen fluid) was used for variable bacteria counts (cellulolytic, proteolytic, and amylolytic) using the roll-tube technique [20].

Blood samples were collected (about 10 mL) from the jugular vein of all experimental animals and then transferred to tubes containing ethylenediaminetetraacetic acid at the same time as the collection of rumen fluid at 0 and 4 hour post morning feeding. Blood samples were quickly put in the refrigerator for 1 hour and then centrifuged for 20 min to separate plasma from the whole blood. Plasma was stored at −20°C for later analysis of blood urea nitrogen (BUN) [21].

Calculation of ruminal CH_4_ production was estimated by using VFA proportions by CH_4_ production = 0.45(acetate, C_2_) −0.275(propionate, C_3_)+0.4(butyrate, C_4_) [22].

### Statistical analysis

All data were statistically analyzed in a 4×4 Latin square design using general linear model procedure [23] according to the model: Y_ijk_ = μ+M_i_+A_j_+P_k_+ɛ_ijk_; where Y_ijk_ = observation from animal *j*, receiving diet *i*, in the period *k*; μ = the overall mean; M_i_ = effect of treatment; A_j_ = effect of animal; P_k_ = effect of period, and ɛ_ijk_ = residual effect. Mean separations with a significantly different value (p<0.05) and difference between treatment means determined by Duncan’s new multiple range test [24]. Trend of Bamboo-Cass level responses was performed by using the orthogonal polynomial.

## RESULTS

### Chemical composition in feed ingredients

Table 1 presents the data of concentrate ingredients and chemical composition of the experimental feeds. The concentrate was formulated using high level of cassava chip (61%) as the main energy source, oil palm meal (20%) and urea (2.5%) as protein and non-protein nitrogen source, respectively. Important concentrations of nutrients such as CP, NDF for concentrate, rice straw, Bamboo grass and Bamboo-Cass were 14.3%, 27.6%; 2.1%, 75.1%; 16.0%, 61.9%; 14.7%, 56.6%, respectively. Plant secondary compounds namely condensed tannins and crude saponins for bamboo grass and Bamboo-Cass were 3.1%, 1.4%; 2.8%, 1.3%, respectively.

### Effect of Bamboo-Cass supplementation on feed intake and nutrients digestibility

Concerning feed intakes (Table 2), as clearly found, rice straw intakes was linearly enhanced (p<0.05) by the supplementation, which raised (p<0.05) the total intake presented as in terms of %BW and g/kg BW^0.75^. Regarding nutrients digestibility of DM, organic matter, CP, NDF, and ADF were increased (p<0.05) when fed the higher level of Bamboo-Cass supplementation.

### Effect of Bamboo-Cass supplementation on rumen fermentation

Rumen parameters are shown in Table 3. The rumen pH (6.7 to 6.8) and temperature (39.2°C to 39.5°C) were in normal range. There were no influences from the dietary treatments on total VFAs (100.4 to 106.8 mmol/L) and C_4_ (10.4% to 10.8%), while the C_2_ and C_3_ concentration, and C_2_:C_3_ ratio were different among treatments (p<0.05). Level of Bamboo-Cass supplementation decreased C_2_, while increased C_3_, as well as the ratio of C_2_:C_3_ (p<0.05). CH_4_ productions (mmol/L) were different by Bamboo-Cass supplementation (p<0.05). Furthermore, ruminal NH_3_-N (mg/dL) concentrations were influenced (p<0.05), while BUN (mg/dL) was similar among treatments.

### Effect of Bamboo-Cass supplementation on microorganism population

Table 4 presents the data of rumen protozoal and bacterial population as influenced by the treatments. Bacterial populations were significantly increased by increasing Bamboo-Cass supplementation, while protozoa populations were linearly reduced (p<0.05). Using roll-tube technique, the rumen amylolytic bacteria (p<0.05) and cellulolytic bacterial populations (p<0.01) were enhanced, while proteolytic count remained unchanged.

## DISCUSSION

### Chemical composition in feed ingredients

The protein content of bamboo grass was 16% of DM, which was within the range of the previous values [9], but higher than the value in literature [7], and lower than the result of previous work [10]. However, condensed tannins and crude saponins contained in bamboo grass was 3.1% and 1.4% of DM which were higher than those in literature [10,11]. The differences occurred could be due to the stage of growth, drying process, harvesting method etc.

### Effect of Bamboo-Cass supplementation on feed intake and nutrients digestibility

The linearly increase of DM intake could be explained with the effect of Bamboo-Cass supplementation that contained additional protein and plant secondary compounds as condensed tannins and crude saponins when fed higher level of Bamboo-Cass. These findings agree with the previous studies [25,26], who reported that supplementation of higher protein with suitable level of fibrous fraction would improve rumen ecology in fermentation and feed degradation, while the cellulolytic bacteria was increased. The digestibility of nutrients would be improved due to ruminal pH, whereas microbial degradation of fiber was inhibited at pH = 6.0 or lower [27]. It enhanced ruminal pH (optimal 6.7 to 6.8) which would increase fiber degradability [28,29]. Furthermore, apparent nutrients digestibilities were affected by Bamboo-Cass supplementation, mainly CP, NDF, and ADF. The decrease of apparent nutrient digestibility of CP was correlated with low NH_3_-N concentration in rumen which could be explained by the effect of condensed tannins in Bamboo-Cass being bound with dietary protein as the protein-tannins complexes which would not degrade in the rumen [30]. However, NDF and ADF were also decreased by supplementation higher level. These could be explained as the effect of tannins could result lower digestibility [31]. The increase of feed intake, feed digestibility, and microbial activity was improved when degradable protein was added [32].

### Effect of Bamboo-Cass supplementation on rumen fermentation

The rumen pH is a key factor to efficient rumen fermentation by activities of microorganisms. The weak acid pH between 6.7-6.9 has been reported the optimal for activity of cellulolytic bacteria namely *Ruminococcus albus*, *Ruminococcus flavefaciens*, *Fibrobacter succinogenes*. Clearly, roughage and concentrate ratio influences ruminal pH and fermentation end-products with a higher supplementation level of concentrate resulting in a higher pH with a higher roughage intake and more C_3_ production [13]. The values of ruminal pH (6.7 to 6.8) and temperature (39.3°C to 39.5°C) were similar and rather stable, which were in the normal range of rumen ecology for fermentation by rumen microbes [28,29]. Effect of Bamboo-Cass on VFA revealed that C_2_ and C_4_ were decreased while C_3_ increased at 150 g/head/d supplementation. This result could be explained that when C_2_ decreased, C_3_ could be increased while the absolute level of C_3_ remained unchanged [33,34]. When acetate to propionate ratio decreased, total VFAs would be decreased with supplementation with high level of dietary condensed tannins [35]. The effect of dietary condensed tannins changed propionate and acetate ratio in the *in vitro* study, by giving the highest propionate and low acetate to propionate ratio [36]. Furthermore, C_2_ inhibitory effect could decrease the C_2_ to C_3_ ratio and inhibiting methanogens in the rumen [37]. This decline of estimated CH_4_ production (p<0.05), resulted by increasing of C_2_ to C_3_ ratio [37], as well as due to decreasing of protozoal population by diets containing condensed tannins [38]. In addition, the formation processes of both propionate and CH_4_ require H_2_, so the increase of propionate could be a competitive pathway with CH_4_ production in the rumen [22]. Supplementation of tannic acid at 6.5, 13.0, and 26.0 g/kg of DM in beef cattle significantly decreased CH_4_ production from 11.1%, 14.7%, and 33.6%, respectively [36]. Tannins could play the role in inhibiting rumen methanogens’ growth and activity by binding with microbial enzymes and proteins as CH_4_ production was decreased [39,40]. Moreover, NH_3_-N concentration was also decreased when supplemented with high level of Bamboo-Cass. This result was similar to the previous study [31] who reported that condensed tannins in the diet were bound by forming protein-tannin complexation, resulting in decreasing protein degradation and NH_3_-N production. Additionally, it was reported that condensed tannins could bind with protein, resulting in lower NH_3_-N concentration [36]. This result could be due to lower NH_3_-N concentration from reducing protein degradability to NH_3_-N in the rumen, hence enhanced protein flowing to intestine [41]. However, there were no effects of saponins on feed intake or digestibility, but saponins were directly affected protozoa numbers and bacterial population by inhibiting the protozoa population and increasing bacterial population resulting a low of methane production when supplementation was at a high level [42].

### Effect of Bamboo-Cass supplementation on microorganism population

Rumen protozoal population decreased, while bacterial population increased, would be due to the effect of condensed tannins and crude saponins in bamboo grass, which has the potential to suppress protozoal growth, as indicated by previous work [43]. Importantly, the increase in bacterial population and the decrease in protozoal population were found when ruminates were fed with the plants containing condensed tannins [40,44]. Moreover, supplementations of Bamboo-Cass, resulted in increasing the amylolytic bacteria, and cellulolytic bacteria while proteolytic bacterial was not affected with 150 g/head/d of Bamboo-Cass supplementation, which could be due to the decrease of protozoal population and hence, the methanogens [45].

## CONCLUSION

Supplementation of Bamboo-Cass can be used as a rumen dietary enhancer as it improved rumen fermentation end-products (C_3_), reduced methane by suppressing protozoal population and increased nutrients digestibility.

## Figures and Tables

**Table 1:** Feed ingredients of concentrate and chemical composition of the feeds

Items	Concentrate	Rice straw	Bamboo grass	Bamboo-Cass
Feed ingredients, % as fresh basis				
Cassava chip	61.0	-	-	-
Coconut meal	12.0	-	-	-
Rice bran	1.0	-	-	-
Palm meal	20.0	-	-	-
Urea	2.5	-	-	-
Molasses	2.0	-	-	-
Sulphur	0.5	-	-	-
Premix1)	0.5	-	-	-
Salt	0.5	-	-	-
Total	100	-	-	-
Chemical compositions				
DM (%)	87.5	88.5	35.7	87.0
	---------------------% of dry matter--------------------			
OM	94.2	87.9	92.5	94.7
CP	14.3	2.1	16.0	14.7
NDF	27.6	75.1	61.9	56.6
ADF	18.2	55.6	40.3	36.7
Condensed tannins	-	-	3.1	2.8
Crude saponins	-	-	1.4	1.3
Minerals (%)				
Calcium	-	-	-	1.5
Phosphorus	-	-	-	0.1
Potassium	-	-	-	2.6
Sodium	-	-	-	0.1
Magnesium	-	-	-	0.4

Bamboo-Cass, Bamboo grass pellet; DM, dry matter; OM, organic matter; CP, crude protein; NDF, neutral detergent fiber; ADF, acid detergent fiber.

1) Contains per kilogram: 4,000,000 IU vitamin A; 400,000 IU vitamin D_3_; 4,000 IU vitamin E; 0.002 g vitamin B_12_; 16 g Mn; 24 g Fe; 10 g Zn; 2 g Cu; 0.05 g Se; 0.2 g Co, 0.5 g I.

**Table 2:** Effect of Bamboo-Cass on feed intake and nutrient digestibility in beef cattle

Items	Bamboo-Cass (g/head/d)				SEM	p-value		
	
	0	50	100	150		L	Q	C
Rice straw DM intake								
kg/d	2.6	3.3	3.4	3.5	0.15	0.087	0.385	0.708
%BW	1.3^a^	1.6^b^	1.7^b^	1.7^b^	0.03	0.025	0.198	1.000
Concentrate intake								
kg/d	1.0	1.0	1.0	1.0	0.01	0.603	1.000	0.315
Total intake								
kg/d	3.6^a^	4.3^b^	4.5^b^	4.7^c^	0.06	0.005	0.367	0.597
%BW	1.8^a^	2.1^b^	2.3^c^	2.3^c^	0.03	0.009	0.186	0.993
Apparent digestibility (%)								
DM	62.5^a^	65.6^b^	66.5^b^	66.1^b^	0.66	0.049	0.425	0.431
OM	71.0^a^	70.0^a^	73.0^b^	72.0^b^	0.49	0.109	0.269	0.047
CP	61.5^a^	63.2^b^	65.0^c^	64.3^b^	0.49	0.833	0.045	0.881
NDF	50.5^a^	52.7^b^	56.6^c^	52.1^a^	0.62	0.150	0.044	0.425
ADF	40.4^a^	43.2^b^	46.3^c^	42.2^b^	0.45	0.201	0.034	0.427
Nutrient intake (kg/d)								
DM	2.6	2.8	3.0	3.0	0.09	0.862	0.525	0.531
OM	2.4	2.7	2.8	2.8	0.08	0.791	0.319	0.283
CP	0.1	0.2	0.2	0.2	0.05	0.236	0.266	0.676
NDF	1.5	1.6	1.9	1.7	0.08	0.697	0.328	0.411
ADF	0.9	1.0	1.2	1.1	0.05	0.617	0.283	0.358

SEM, standard error of mean; L, linear; Q, quadratic; C, cubic; Bamboo-Cass, Bamboo grass pellet; DM, dry matter; BW, body weight; OM, organic matter; CP, crude protein; NDF, neutral detergent fiber; ADF, acid detergent fiber.

a–c Values within the row with different superscripts are significantly different (p<0.05).

**Table 3:** Effect of Bamboo-Cass on rumen fermentation in beef cattle

Items	Bamboo-Cass (g/head/d)				SEM	p-value		
	
	0	50	100	150		L	Q	C
Rumen pH								
0 h-post feeding	6.7	6.8	6.7	6.8	0.02	0.245	0.544	0.091
4	6.7	6.7	6.6	6.7	0.02	0.591	0.291	0.093
Mean	6.8	6.8	6.7	6.8	0.02	0.220	0.719	0.056
Temperature (°C)								
0 h-post feeding	39.2	39.2	39.2	39.3	0.11	0.645	0.730	0.877
4	39.5	39.3	39.3	39.2	0.07	0.345	0.242	0.291
Mean	39.3	39.3	39.2	39.2	0.06	0.750	0.228	0.058
NH_3_-N (mg/dL)								
0 h-post feeding	14.2^a^	15.6^b^	19.5^c^	13.5^a^	0.44	0.664	0.006	0.020
4	13.5^b^	16.7^b^	13.5^b^	13.2^a^	0.22	0.072	0.008	0.003
Mean	13.9^a^	16.2^b^	16.5^b^	13.4^a^	0.20	0.519	0.001	0.442
BUN (mg/dL)								
0 h-post feeding	13.0	13.0	13.5	13.5	0.14	0.172	1.000	0.468
4	13.8	14.0	14.0	14.0	0.21	0.708	0.779	0.900
Mean	13.4	13.5	13.8	13.8	0.17	0.396	0.859	0.812
Total VFA (mmol/L)								
0 h-post feeding	99.9	106.2	101.2	101.8	1.92	0.971	0.482	0.358
4	100.9	107.3	108.2	105.3	1.55	0.352	0.189	0.908
Mean	100.4	106.8	104.7	103.6	1.60	0.625	0.291	0.537
Acetate (C_2_) (%)								
0 h-post feeding	74.8	70.2	72.1	70.4	0.62	0.158	0.336	0.127
4	74.3^b^	66.1^a^	67.9^a^	67.5^a^	0.76	0.034	0.042	0.124
Mean	74.5^b^	68.2^a^	69.6^a^	68.9^a^	0.47	0.010	0.022	0.054
Propionate (C_3_) (%)								
0 h-post feeding	14.4	18.2	16.6	18.5	0.61	0.249	0.705	0.184
4	15.1^a^	23.7^b^	22.7^b^	22.4^b^	0.72	0.018	0.019	0.147
Mean	14.7^a^	21.0^c^	19.3^b^	20.3^b^	0.56	0.021	0.052	0.165
Butyrate (C_4_) (%)								
0 h-post feeding	10.8	11.6	11.4	11.1	0.41	0.594	0.357	0.659
4	10.6	10.1	9.4	10.1	0.13	0.128	0.069	0.205
Mean	10.7	10.8	10.4	10.6	0.24	0.739	0.920	0.582
C_2_:C_3_ ratio								
0 h-post feeding	5.2^c^	3.9^b^	4.4^b^	3.8^a^	0.18	0.048	0.508	0.110
4	4.9^b^	2.8^a^	3.0^a^	3.0^a^	0.17	0.011	0.018	0.144
Mean	4.4^c^	3.4^a^	3.8^b^	3.5^a^	0.11	0.044	0.176	0.049
Estimated methane production (mmol/100 mol)								
0 h-post feeding	34.0^c^	31.2^a^	32.4^b^	31.0^a^	0.33	0.032	0.169	0.048
4	33.5	27.3	28.1	28.3	0.59	0.103	0.058	0.206
Mean	33.8^c^	29.3^a^	30.3^b^	28.9^a^	0.32	0.003	0.051	0.033

SEM, standard error of mean; L, linear; Q, quadratic; C, cubic; Bamboo-Cass, Bamboo grass pellet; NH_3_-N, ammonia nitrogen; BUN, blood urea nitrogen; VFA, volatile fatty acid; C_2_:C_3_, acetate:propionate.

a–c Values within the row with different superscripts are significantly different (p<0.05).

**Table 4:** Effect of Bamboo-Cass on microbial population in beef cattle

Items	Bamboo-Cass (g/head/d)				SEM	p-value		
	
	0	50	100	150		L	Q	C
Total direct count (cells/mL)								
Protozoa (×10^5^)								
0 h-post feeding	2.8	2.6	2.6	2.4	0.11	0.283	0.779	0.708
4	3.9	2.3	2.3	2.3	0.31	0.161	0.278	0.613
Mean	3.3^b^	2.4^a^	2.4^a^	2.3^a^	0.18	0.049	0.369	0.617
Bacteria (×10^11^)								
0 h-post feeding	28.0	28.3	28.4	28.6	0.18	0.495	0.078	0.072
4	30.1^a^	31.4^a^	35.6^b^	37.5^c^	0.43	0.001	0.077	0.965
Mean	29.6^a^	29.9^a^	32.0^b^	33.0^c^	0.29	0.003	0.066	0.501
Roll-tube technique (CFU/mL)								
Amylolytic (10^8^)								
0 h-post feeding	3.1^a^	3.7^b^	4.3^c^	3.1^a^	0.14	0.738	0.020	0.200
4	4.0^a^	5.7^b^	5.8^b^	6.8^c^	0.17	0.002	0.325	0.146
Mean	3.6^a^	4.7^b^	5.1^b^	4.9^b^	0.14	0.013	0.069	0.809
Proteolytic (10^8^)								
0 h-post feeding	2.3	2.4	1.9	2.2	0.09	0.417	0.654	0.137
4	3.2^b^	2.7^a^	2.9^a^	3.1^b^	0.06	0.077	0.004	0.148
Mean	2.7	2.6	2.4	2.7	0.10	0.675	0.363	0.638
Cellulolytic (10^9^)								
0 h-post feeding	4.8	4.8	4.9	5.5	0.10	0.057	0.204	0.692
4	4.8^a^	7.2^b^	8.0^c^	9.8^d^	0.14	0.001	0.322	0.089
Mean	4.8^a^	6.0^b^	6.4^b^	7.6^c^	0.12	0.001	0.831	0.243

SEM, standard error of mean; L, linear; Q, quadratic; C, cubic; Bamboo-Cass, Bamboo grass pellet; CFU, colony-forming unit.

a–d Values within the row with different superscripts are significantly different (p<0.05).
